# Doctors (Poem)

**Published:** 1875-07

**Authors:** Will Carleton


### *DOCTORS.*

Good folks ever will have their way,
Good folks ever for it must pay:
But we, who are here and everywhere,
The burden of their faults must bear.
We must shoulder others' shame,
Fight their follies and take their blame;
Purge the body and humor the mind;
Doctor the eyes when the soul is blind;
Build the column of health erect
On the quicksands of neglect;
Always shouldering others' shame—
Bearing their faults and taking the blame.

WILL CARLETON.

